# Sexual behavior and sexually transmitted diseases among the female partners of inmates*

**DOI:** 10.1590/1518-8345.2568.3043

**Published:** 2018-10-11

**Authors:** Debora Cristina Martins, Giovanna Brichi Pesce, Giordana Maronezzi da Silva, Carlos Alexandre Molena Fernandes

**Affiliations:** 1 Universidade Estadual de Maringá, Programa de Pós-graduação em Enfermagem, Maringá, PR, Brazil.; 2 Centro Universitário de Maringá, Centro de Ciências da Saúde, Maringá, PR, Brazil.; 3 Prefeitura Municipal de Apucarana, Autarquia Municipal de Saúde, Apucarana, PR, Brazil.; 4 Universidade Estadual do Paraná, Faculdade Estadual de Educação Ciências e Letras de Paranavaí, Paranavaí, PR, Brazil.

**Keywords:** Sexually Transmitted Diseases, Sexual Behavior, Women’s Health, Prevention and Control, Vulnerable Populations, Nursing

## Abstract

**Objective:**

to analyze the sexual behavior of the female partners of inmates and estimate the prevalence of sexually transmitted diseases.

**Method:**

cross-sectional, quantitative study involving 349 female partners of inmates. The *Estudo de Comportamento Sexual* [Sexual Behavior Study], an instrument validated in Brazil, was used to collect the data. The Statistical Package for the Social Sciences, version 20 was used in the statistical analysis.

**Results:**

41.2% of the female partners of inmates reported a prior history of sexually transmitted disease. Association was found between having more than one partner in the last 12 months (<0.006), sexual violence (<0.001), having sex for money (<0.001), under the influence of alcohol (<0.001), and under the influence of drugs (<0.005). The variables associated with sexually transmitted infections in the logistic regression were: having more than one partner in the last 12 months, sexual violence, sex for money, and under the effect of alcohol or drugs.

**Conclusion:**

The number of partners, sexual violence, sex for money, and under the influence of alcohol or drugs are sexual risk behaviors that increase the prevalence of sexually transmitted infections among the female partners of inmates.

## Introduction

The increased prevalence, frequent occurrence and consequences of Sexually Transmitted Diseases (STD) among women reveal the need to address these issues from a gender perspective. Biological, cultural, and socioeconomic factors contribute to the high incidence and prevalence of STD and infection by Human Immunodeficiency Virus (HIV) among women^1^. A large share of women, in particular those subject to greater social vulnerability do not adopt protective measures against STD, due to difficult access to preventive devices and services, lack of knowledge, gender issues and/or unstable relationships. More than 20 types of diseases are transmitted through sexual contact and represent a severe public health problem given their consequences for health and social and economic repercussions^1^ ^-^ ^2^.

STD are contagious infections, the most frequent form of transmission is through sexual intercourse (mainly through vaginal, oral or anal sex). They are caused by various infectious agents and cause a large range of symptoms and clinical manifestations, though, in most cases, these conditions may progress with few or no symptoms^2^. Currently, STD are a public health problem worldwide, imposing increasing socioeconomic costs, not only because of the high number of infected individuals but also because of their increased incidence in many countries, and more importantly, because of the consequences for sexual, reproductive and maternal-fetal health, and ease with which infections are acquired and transmitted. It is, however, difficult to establish a single sexual risk behavior^3^. 

Populations with a history of incarceration and their sexual partners are exposed to a high risk when compared to populations not exposed to incarceration. Behaviors such as having multiple partners, concomitant partners, and unprotected sex predispose and influence the risk of STD, however, vulnerability, social and economic instability and substance use, such as alcohol and drugs, also contribute to increased risk among women with imprisoned partners. The following stand out among factors that potentially determine the transmission of these diseases, which suggest high vulnerability: irregular or infrequent use of condoms, low educational level, multiple sexual partners, sex under the influence of alcohol and/or drugs, sexual violence, sex for money, and low involvement with preventive measures^4^. 

The environment of prisons offers physical and psychological risks and the risk of infectious diseases due to the heterogeneity of incarcerated individuals^5^. In this sense, the vulnerability of both prisoners and their family members, especially that of their partners, should be taken into account and be a priority in the planning of care actions considering the risk behaviors of this population. Some factors related to vulnerability include lack of access to information and educational activities, addressing how STDs/HIV are transmitted and prevented; lack of motivation or sensitization to assess and understand the risk of infections to which they are exposed; lack of skills to adopt preventive measures including safer life habits^1^. In relation to the female partners of inmates, it is necessary to become familiar with the National Comprehensive Care Policy on Persons Deprived of Liberty (PNAISP). One of its objectives is to consider the health/disease/care delivery continuum beyond the individual, including his family and social networks^6^.

Actions implemented in Brazil to prevent and control STD/HIV primarily focus on the use of condoms in every sexual intercourse. Approaches recommending decreasing the number of partners, sexual abstinence or monogamy, are unfeasible or unviable and disrespect the right of people to decide when and with whom they keep sexual intercourses, thus, these approaches are not part of the preventive strategies implemented in Brazil^7^. Addressing the various sexual practices, highlighting male and female (biological and gender-related) vulnerabilities is essential for men and women to perceive the risk situations they are exposed to, not only considering their own sexual behavior, but also that of their partners^8^.

Brazil is the fourth country in the world with the highest number of prisoners - there are currently 623,000 inmates serving sentences in prisons and precincts of police stations, and this number tends to increase by 7% a year^9^. With this high number of inmates and progressive increase every year, the estimate is that thousands of women have intimate visits with their partners and are considered a vulnerable population, exposed to risks and behaviors that may result in SDT or other diseases. 

Given the previous discussion and hypothesis concerning the condition of vulnerability and types of sexual risk behavior that may be associated with the prevalence of STD among the female partners of inmates, this study’s objective was to analyze sexual behavior of the female partners of prisoners and the prevalence of STD. There are few studies addressing the female partners of inmates in Brazil so that these results can encourage future studies and support the actions of health services, favoring the development of necessary prevention and health promotion measures, in addition to continued care directed to the health of these women as well that of their imprisoned partners.

## Method

This cross-sectional quantitative study was conducted with 349 female partners of inmates from the three largest penitentiaries of the state of Paraná, Brazil from January to July 2016. A convenience sample was collected from each of the three largest penitentiaries of the state of Paraná with a closed prison system and male offenders: the first prison belongs to the 1^st^ Regional located in Piraquara. The city of Piraquara is located in the South of the state of Paraná, with a population of 102,798 inhabitants according to IBGE (Brazilian Institute of Geography and Statistics), and currently houses the largest penitentiary complex of the state with approximately 1,635 male inmates^9^ ^-^ ^10^.

The second penitentiary is located in the North of the state and belongs to the city of Londrina, located in the South of Brazil, with a population of 543,003 inhabitants according to IBGE. It is the second most populated city in the state and the fourth in the South, currently housing the second largest complex of the state with approximately 1,150 inmates^9^ ^-^ ^10^.

The third penitentiary is located in the Southeast of the state and belongs to the 7^th^ Regional in the city of Francisco Beltrão. The city of Francisco Beltrão is located in the Southeast of the state of Paraná with a population of 85,486 inhabitants, according to the IBGE’s estimates. It houses the third largest penitentiary complex in the state with approximately 1,135 inmates^9^ ^-^ ^10^.

According to reports of prison agents and the Paraná Department of Public Security, approximately 80% of inmates are visited by their partners and can have intimate visits according to a monthly schedule established to meet the high demand^9^. The penitentiary of the city of Piraquara receives approximately 900 women, the penitentiary in the city of Francisco Beltrão receives approximately 600 women, and the city of Londrina receives about 700 women (n=2,200).

Considering a confidence level of 95%, maximum error of 5%, 50% population share, and 10% margin to account for potential losses, stratified sampling with proportionate allocation resulted in a sample size of 366 women distributed across the state’s three penitentiaries. Thus, 136 women composed the sample in the city of Piraquara, 74 women in the city of Francisco Beltrão, and 139 women in the city of Londrina. Each of the 366 questionnaires was revised. Nine were excluded because only the identification form was completed, and another eight questionnaires because more than 20.0% of the questions had not been completed in the instrument *Estudo de Comportamento Sexual no Brasil (Ecos)* [Sexual Behavior Study], totaling 349 valid questionnaires (95% of the calculated sample).

Women older than 18 years old, partners of inmates, who had made intimate visits to their partners for more than six months and voluntarily provided their consent, participated in this study. Women with other levels of kinship (e.g., mothers, daughters and others) and those who were under the influence of alcohol or other drugs were excluded.

The women were randomly selected on the days and hours scheduled for intimate visitation with their partners in the penitentiaries. Data collection was conducted in the prison’s patios where women waited for their visits, ensuring that the interviews were private and remained confidential.

In order to establish rapport with the participants, the World Health Organization Quality of Life (WHOQOL-bref) was applied first. It is an instrument with 26 questions addressing quality of life, health and others spheres of life. The first question refers to general quality of life and the second question to satisfaction with one’s own health. The other 24 questions are divided into the physical, psychological, social relationships and environment domains. It is an instrument that can be applied to both healthy populations and populations affected by chronic diseases. In addition to its cross-cultural nature, this instrument assesses the individual perception of people and can assess quality of life in diverse groups and situations^12^.

Afterwards, the semi-structured questionnaire Ecos, model II, with 38 questions was applied. It was adapted for application in a field survey addressing only women. The questionnaire Ecos was developed by Abdo and collaborators in three models, models I, II and III. Model II was modified and applied with guiding questions directed to the population of female partners of inmates and considering their sociodemographic profile. The merit of this instrument was not only revealing data on sexual risk behavior but also portraying different aspects and providing a profile of the current and past sexual behavior of the population under study^13^.

The first part of this instrument is intended to portray the profile and sociodemographic characteristics of the participants, as well as to screen for risk factors related to lifestyle (consumption of alcohol, tobacco, illegal drugs and physical exercise). The second part of the instrument is intended to identify STD, asking whether they had some type of STD diagnosed by a physician currently or in the past, as well as types of sexual behavior (age of the first sexual intercourse, number of partners in the last 12 months, sex under the influence of alcohol, sex under the influence of drugs, sex for money, sexual violence). The participants should answer yes or no. 

Data were entered in an Excel worksheet, Windows 2007 and later statistically analyzed using Statistical Package for the Social Sciences (SPSS), version 20. The Kolmogorov-Smirnov test, graphic methods, and standardized values of asymmetry and kurtosis (±2*Z*) were used to identify normality of data. 

To characterize the sample, descriptive statistics included absolute and relative frequency for the categorical variables, as well as median and interquartile interval for the continuous variable (when sexual life was initiated) due to its non-parametric distribution. Chi-square was used to verify differences in the proportions between dependent variable (STD) and independent variables. Yacht Continuity Correction was performed in 2x2 contingency tables.

Multivariate Logistic Regression was used to determine Odds Ratio (OR) and respective Confidence Intervals (CI) (95%), aiming to analyze association of STD with independent variables. The criterion applied to include independent variables in the multivariate model was level of association of p≤0.20 with the dependent variable, using Chi-square, later presented in the model with p≤0.05. 

The study was authorized by the three prison institutions and later by the Brazilian Penitentiary Department (Depen) in Paraná. All ethical and legal guidelines established by Resolution 466/2012, CNS - MS concerning research with human subjects were complied with and the Institutional Review Board at School of Apucarana (FAP) approved the study (Report opinion 1,330,747. Invitation to participate in the study was accompanied by two copies of free and informed consent forms containing information regarding the study’s objectives, type of participation and interview method. The interviewees kept one copy and the interviewer kept another.

## Results

From the total sample (n=349), 39.0% (n=136) were from Piraquara; 39.8% (n=139) from Londrina; and 21.2% (n=74) from Francisco Beltrão. Most women (51.9%) were aged between 20 and 29 years old. With regard to race, Caucasian and mixed race were the most frequently mentioned, 41.5 and 42.1%, respectively. With regard to marital status, 49.0% reported a stable relationship, that is, they lived with their “partners” but were not officially married; 21.2% were single; and 29.8% were married. The number of children was also verified; more than half (53.3%) had one or two children. The dependent variable STD stands out as 41.2% of the women reported they currently had or previously had some type of STD. Finally, the median age these women initiated sexual life was 14 years old.

Table 1 presents the proportions of categories of independent variables with the proportion or dependent variable (STD). Variables associated with the presence of SDT were: having more than one partner in the last 12 months (p<0.006); sexual violence (p=<0.001); sex for money (p=<0.001); sex under the influence of alcohol (p=<0.001); and sex under the influence of drugs (p=<0.001). 

**Table 1:** Distribution of the population of the female partners of inmates and sexual behavior in terms of sexually transmissible diseases. Piraquara, Londrina, Francisco Beltrão, Paraná (PR), Brazil, 2016 (n=349)

Variables		Total		STD*		p-value^†^
		N	(%)	N	(%)	
Age	<30 years old	231	66.4	97	66.0	0.894
	≥30 years old	117	33.6	50	34.0	
Education	Inc Prim Edu.^‡^	109	31.3	50	34.5	0.736
	Prim Edu^§^	37	10.6	14	9.5	
	Inc. high^││^	111	31.9	45	30.6	
	High school^¶^	60	17.2	28	19.0	
	Some college	22	6.3	7	4.8	
	College^††^	9	2.6	3	2.0	
Partner/12 months	1	206	59.2	74	50.3	<0.006^†^
	>1	142	40.8	73	49.7	
Uses condoms	Never	198	59.6	78	53.1	0.295
	Sometimes	143	41.1	67	45.6	
	Always	7	2.0	2	1.4	
Sexual violence	Yes	111	31.9	84	57.1	<0.001^†^
	No	237	68.1	63	42.9	
Sex for money	Yes	79	22.7	53	36.1	<0.001^†^
	No	269	77.3	94	63.9	
Alcohol	Yes	211	60.6	106	72.1	<0.001^†^
	No	137	39.4	41	27.9	
Drugs	Yes	71	20.4	41	27.9	0.005^†^
	No	277	79.6	106	72.1	

*STD: reports of Sexually Transmitted Diseases; †Chi-square; statistically significant p≤0.05; ‡Incomplete primary/middle education; §Complete Middle Education; ││Incomplete high-school; ¶Complete high-school; **Some college studies; ††College degree.

Using robust logistic regression, Table 2 shows chances of agreement with the categories of variables, in separate, regardless of STD, and the associated variables were the same variables that appeared associated according to the Chi-square analysis (Table 2). Women who had sexual intercourse with more than one partner in the last 12 months were 1.9 times (CI95% 1.2-3.0) more likely to acquire STD. Women who reported sexual violence were 8.6 times (CI95% 5.1-14.7) more likely; those who received money for sex were 3.8 times (CI95% 2.2-6.5); those who had sex under the influence of alcohol were 2.3 times (CI95% 1.5-3.7); and under the influence of drugs women were 2.2 times (CI95% 1.3-3.7) more likely to acquire SDT regardless of having a prior diagnosis of SDT. The variables that appeared in the adjusted regression were: sexual violence 7.4 (CI95% 4.3-12.7) and sex for money 2.5 (CI95% 1.3-5.0).

**Table 2:** Distribution of factors associated with sexually transmitted infections in the population of female partners of inmates, measured through Logistic Regression. Piraquara, Londrina, Francisco Beltrão, Paraná (PR), Brazil, 2016 (n=349)

Variables		Raw Odds ratio	Adjusted Odds ratio
		(CI*95%)	(CI*95%)
Age	<30 years old	1	-
	≥30 years old	1.0 (0.71.6)	-
Education	College^‡^	1	-
	Inc. Prim.^§^	1.3 (0.4-4.0)	-
	Prim. Edu.^││^	1.8 (0.7-4.8)	-
	Inc. high^¶^	1.9 (0.7-5.2)	-
	High-school**	1.4 (0.5-3.9)	-
	Some college^††^	1.0 (0.2-5.9)	-
Partners/12 months	1	1	1
	>1	1.9 (1.2-3.0)^†^	0.8 (0.4-1.5)
Use of condoms	Always	1	-
	Sometimes	2.2 (0.4-11.8)	-
	Never	1.6 (0.3-8.6)	-
Sexual violence	No	1	1
	Yes	8.6 (5.1-14.7)^†^	7.4 (4.3-12.7)^†^
Sex for money	No	1	1
	Yes	3.8 (2.2-6.5)^†^	2.5 (1.3-5.0)^†^
Alcohol	No	1	1
	Yes	2.3 (1.5-3.7)^†^	1.7 (0.9-3.0)
Drugs	No	1	1
	Yes	2.2 (1.3-3.7)^†^	1.0 (0.5-2.0)

*CI: Confidence Interval; †p≤0.05 (p-value - Chi-square test for statistically significant differences according to raw odds ratio and adjusted odds ratio); ‡College degree; §Incomplete Primary/middle Education; ││Complete middle education; ¶Incomplete high-school; **Complete high-school; ††Some college studies.

## Discussion

This study’s results show a high concentration of STD among the female partners of inmates as more than 41% of the participants reported prior or current STD. Additionally, the findings reveal there is a relationship between STD among the female partners of inmates and education, number of partners, sex for money, and the consumption of alcohol and/or drugs; these variables significantly increased the likelihood of acquiring such diseases in the population under study.

Note that this population composed of female partners of inmates presented low educational level; 74.6% had not completed high school. They also experience social vulnerability as low education is a characteristic of individuals under social vulnerability, who may be more predisposed to diseases, as statistical data provided by the Brazilian Ministry of Health show^14^. Note that the type of sexual behavior reported by the female partners of inmates may be associated with a low educational level and social vulnerability, as these aspects are directly linked to information access, negatively influencing sensitization and understanding on how to prevent and treat certain diseases^15^. 

This study’s results show that 40.8% of the women reported more than one sexual partner in the last 12 months, and 49.7% of them reported a prior STD. This result contrasts with that reported in a study conducted with 175 female partners of inmates in the United States, which revealed that 50% of the women reported having other sexual partners while their partners were incarcerated. Men with a history of incarceration are three to six times more susceptible to acquire HIV and other STD than men with no history of incarceration^16^. It poses increased risk to the female partners of inmates because, in addition to the risks these women are individually exposed, there is the sexual risk behavior of their incarcerated partners^17^. 

Women whose partners are imprisoned lack the emotional and material support a present partner provides in the family context. This lack of support and resources may lead these women to seek another partner to fill in the gaps left by their imprisoned partners^16^ ^,^ ^18^. Thus, the participants consider this behavior to be acceptable, especially in situations in which their partners may receive resources in exchange for sexual intercourse within the prison^16^. Researchers conclude that women who engaged in other relationships when their partners were incarcerated were more likely to be young and engage in other sexual relationships and present risk behaviors such as the use of alcohol and drugs^18^.

This study also shows that 36.1% of the female partners of inmates who reported receiving money for sex currently have or have had STD and were 3.8 times more likely to acquire SDT. A longitudinal study using a qualitative approach and addressing the female partners of Afro-American inmates highlight that the involvement of these women with other partners is also associated with financial issues, as they attempt to ensure shelter and support for their families^4^. Additionally, there are factors directly associated with SDT among women who make sex for money, such as a high number of sexual partners and unprotected sex together with the consumption of illegal drugs and alcohol, exposure to prisons, low education and socioeconomic marginalization^7^ ^,^ ^19^.

With regard to risk behavior for SDT, note that in addition to the number of partners and making sex for money, there is also the fact that sexual intercourse takes place under the influence of alcohol and drugs. This study’s findings show that women who reported sexual practice under the influence of alcohol were 2.3 more likely to contract SDT while those under the influence of drugs were 2.2 more likely. Some positive expectations, on the part of users concerning the effect of alcohol, are associated with a greater propensity to have unprotected sex after drinking. Additionally, alcohol decreases one’s perception of risk and hinders decision-making. One of the greatest problems arising from such behavior is having unprotected sex, which increases the risk of acquiring STD^20^.

A study conducted with 673 women in Africa conclude, through a control group, that women who consumed alcohol were more likely to have multiple partners and partners who presented a risk behavior. Generalized estimating equation models for binary outcomes show the impact of alcohol at the beginning of the study addressing sexual risk behavior and SDT during a period of 12 months. Women who consumed alcohol tested positive for chlamydia and other STD when a vaginal swab test was performed and also reported not using condoms with casual partners during a 12 month-follow-up^20^.

To investigate the practice of sex under the influence of drugs, researchers conducted a study in the South of Brazil using a convenience sample and tested for HIV and other SDT. They addressed 258 female adolescents and a rate of 7.4% of HIV seropositivity was found. Multiple analyses including two composed variables show that the use of drugs was associated with positive HIV and other STD, corroborating the results of this study^21^. Drugs are believed to cause uninhibitedness and increase sexual desire, leaving individuals more prone to sexual risk practices^21^. Additionally, many people use drugs to relax in order to reach out to someone they intend to establish a relationship with and individuals prone to risk behaviors may concomitantly use alcohol and other drugs^22^.

Note that sexual violence appears very influential in this study and is an aspect that also presents risk with regard to STD, as 57.1% of the women who experience sexual violence also acquired some type of STD. Such information shows that women in a situation of violence cannot even protect themselves or choose sexual behaviors as they submit themselves out of fear and are forced to practices that expose them to such diseases. A situation that is common among women, victims of sexual violence, is to submit to marital sex without desire, which may be associated with the fact that women may not perceive unconsented sexual intercourse as violence and become vulnerable to STD/HIV^23^. The consequences of sexual violence go beyond the physical and psychological spheres, as these women are directly exposed to an “undesirable pregnancy, STD, HIV/AIDS infection, depression, panic syndrome, anxiety and psychosomatic disorders”^24^. The aggressions experienced by the female partners of inmates may be linked to alcohol and other drugs abuse because, when their partners are not under the influence, their behavior may be calmer^21^. Additionally, the use of alcohol and drugs may lead a man to force his partner to practice sex, aggravating cases of violence even further^23^. Note that these factors contribute to sexual violence and appear very frequently in this study’s results.

Therefore, we highlight the importance of providing integral care to the health of the female partners of inmates, which represents a challenge for the health system, especially in the different facilities where care is provided. It is crucial to take into account the habit of consuming alcohol and using illegal drugs that may contribute to sexual risk behavior, as well as number of partners, sexual violence, and making sex in exchange for money, which expose women to unprotected sex, and consequently, STD. Thus, this approach needs to be considered as part of the routine implemented in health services, especially in primary health care services^25^. Actions to promote the health and civil rights of the female partners of inmates is essential for these women who become invisible for the judicial and health systems, which only focus on the improved behavior of inmates when allowed to have intimate visitations, not taking into account the psychological conditions and specific health conditions of women, or STD prevention and treatment when necessary^5^ ^,^ ^26^.

## Conclusion

This study’s results show association of highly risk sexual behavior for the emergence of sexually transmitted diseases among the female partners of inmates. These behaviors are closely linked to number of sexual partners, having sex for money and under the influence of alcohol and drug. It is clear that women who have a relationship with inmates are vulnerable to STD and require differentiated care to prevent and treat diseases when necessary.

Nevertheless, the dynamics of healthcare delivered within prisons mainly has a curative and eventually preventive nature. There is much to be done in order to consolidate the promotion and preservation of health in primary healthcare services, emphasizing on the importance of health education. Additionally, the routine of the female partners of inmates, the knowledge they acquire through experience, how they understand health, their practices toward health and their sexual behavior need to be known in order to meet their health needs, which encourage new studies and the creation of new strategies in the field of heath care delivery within the Brazilian Public Health System (SUS), with the objective to include popular understanding and ways to care for health.

The results reveal that strategies to promote, prevent and implement interventions addressing sexual health within the prison environment should comprise the complexity of peculiarities the female partners of inmates experience. Strengthening the autonomy of these women, as the essence of an education process, in addition to taking into account science, knowledge and opinions, should include the context of environmental, social and cultural contexts.

This is one of the few studies addressing this population. Further research is recommended to contribute with improved scientific evidence that concerns this population in Brazil, considering the large number of women inside penitentiaries and prisons who frequently visit their partners.
